# Evaluation of current practice of antimicrobial use and clinical outcome of patients with pneumonia at a tertiary care hospital in Ethiopia: A prospective observational study

**DOI:** 10.1371/journal.pone.0227736

**Published:** 2020-01-30

**Authors:** Theodros Fenta, Ephrem Engidawork, Wondwossen Amogne, Alemseged Beyene Berha

**Affiliations:** 1 Department of Pharmacology and Clinical Pharmacy, School of Pharmacy, College of Health Sciences, Addis Ababa University, Addis Ababa, Ethiopia; 2 Department of Infectious Diseases, School of Medicine, Addis Ababa University, Addis Ababa, Ethiopia; Columbia University, UNITED STATES

## Abstract

**Background:**

Antimicrobial resistance, which is commonly observed in the management of pneumonia, is a major threat to public health and is driven by inappropriate antimicrobial use. The aim of this study was therefore to assess the current practice of antimicrobial utilization and clinical outcomes in the management of adult pneumonia at Tikur Anbessa Specialized Hospital.

**Method:**

A prospective observational study was conducted in the internal medicine wards of Tikur Anbessa Specialized Hospital. The study was conducted from 1 September 2016 to 30 June 2017 and patients aged ≥ 14 years and diagnosed with pneumonia were included. Chart review and self-administered questionnaire were used to collect data regarding pneumonia diagnosis and management as well as clinical outcomes (stable, complications, and in-hospital mortality). Descriptive statistics and binary logistic regressions were performed for data analyses.

**Results:**

Out of 200 enrolled patients, clinical diagnosis was supported by microbiologic testing and imaging in 75 (37.5%) and 122 (61.0%) cases, respectively. The treatment approach in almost all patients (99.5%) was empirical and no de-escalation therapy was made even after acquiring culture results. The total duration of antimicrobial therapy was 12.05±5.09 days and vancomycin was the most commonly prescribed antimicrobial agent (25%), with 70% of the patients receiving this drug empirically. Nearly, 30% of the patients missed their antimicrobial doses during the course of treatment and stock-out (36.7%) was the major reason. Close to 113 (66%) of the treating physicians used reference books to prescribe antimicrobial agents. Patients’ outcomes were found to be stable (66%), in-hospital mortality (18.5%), and ending up in complications (17%). Poor clinical outcome (death and complicated cases) was found to be associated with recent antimicrobial use history (p = 0.007, AOR 2.86(1.33–6.13)), cancer (p = 0.023, AOR 3.46(1.18–10.13)), recent recurrent upper respiratory tract infection (p = 0.046, AOR 3.70(1.02–13.40)), respiratory rate >24 breaths/min or <12 breaths/min (p = 0.013, AOR 2.45(1.21–4.95)) and high level of serum creatinine after initiation of antimicrobial therapy (>1.4mg/dl) (p = 0.032, AOR 2.37(1.07–5.20)).

**Conclusion:**

Antimicrobials are empirically prescribed without sufficient evidence of indication and microbiological or radiological findings. The practice also is not based on local guidelines and no multidisciplinary approach is apparent. [How about: “It is likely that these factors contributed to higher rates of mortality (18.5%) when compared with similar studies in other countries” instead of this “As a result, there were higher rates of mortality (18.5%) when compared with other similar studies”]. Hence, the hospital requires a coordinated intervention to improve rational use of antimicrobials and clinical outcomes through establishing an antimicrobial stewardship program.

## Background

The introduction of antimicrobials, since the discovery of penicillin in the early 1940s, has been a critical component of public health in saving lives of millions of people worldwide [1]. However, the successful use of antimicrobial agents is compromised by misuse [2] and development of resistance [3] in the past few years.

Inappropriate antimicrobial use is a major driver of antimicrobial resistance (AMR) [4]. Earlier studies conducted in the same tertiary care hospital reported inappropriate use of antimicrobials, particularly cephalosporins [5]. Moreover, such practices have been repeatedly observed during multi-disciplinary as well as pharmacist-led ward rounds on pneumonia patients. [6]. Pneumonia is one of the leading causes of morbidity and mortality [7]. In recent years, hospitalization due to pneumonia has been increasing in elderly patients as well as those with multiple co-morbidities [8, 9].

The possible consequences of inappropriate antimicrobial use include toxicity, emergence of antimicrobial resistance, hospital-acquired infections (HAIs), increased morbidity and mortality, prolonged hospitalization, and increased health care expenditures [10–12]. Although there is a constant need for new antimicrobials to circumvent infectious disease challenges, many companies are abandoning or shifting away from antimicrobial development [13].

Reports indicate that there would be about 10 million AMR related deaths every year until 2050, with the majority being in Africa and Asia [14]. The Ethiopian Federal Ministry of Health and the Food, Medicine, and Healthcare Administration and Control Authority (FMHACA) have been working in concert to combat AMR through developing national drug policies [15] and treatment guidelines [16]. FMHACA also developed a national strategic framework for the prevention and containment of AMR in 2011. The objective is to tackle AMR through promotion of rational antimicrobial use, infection control & surveillance, and strengthening research & education in the country [17]. These efforts are made to promote rational use of antimicrobials and ultimately to safeguard citizens. However, effective implementation of the standards and most of the treatment guidelines are not yet studied.

The present study was therefore initiated to evaluate antimicrobial use patterns and clinical outcome in the management of pneumonia. The findings could help policymakers to design appropriate intervention strategies so that antimicrobial utilization could be optimized and patient and economic outcomes are improved.

## Materials and methods

### Ethics statement

The proposal including Amharic written verbal consent, which was attached as an annex, was submitted to the School of Pharmacy, Addis Ababa University ethical review committee for review and approval. The study was conducted after securing the letter of ethical approval (ESR/SOP/88/06/2016). Verbal consent from patients was obtained after the provision of information regarding the purpose of the study. Patients were told the reasons of being selected to be included in the study and assured that declining participation would not have any influence on the right to get treatment. Patients were also told about their rights to withdraw from the study at any time. Participants were assured about confidentiality (privacy and anonymity) of the information obtained in the course of the study.

### Study area

Tikur Anbessa Specialized Hospital (TASH) is a tertiary care teaching hospital in Addis Ababa, Ethiopia, with over 700 beds. The data was collected from the internal medicine wards, which have around 95 beds. Based on the 2016 health management information system (HMIS) data of the hospital, annual patient visits were around 500,000, out of which admission to internal medicine wards accounted for 2100 patients.

### Study design

A prospective observational study was conducted in adult patients with pneumonia admitted to the internal medicine wards.

### Data collection procedure

Data collection instruments (data abstraction format and self-administered questionnaire) were developed through mining of the literature on antimicrobial utilization, antimicrobial resistance, and antimicrobial stewardship guidance. The data abstraction format was designed to help extract information on patient socio-demographic and clinical characteristics. The self-administered questionnaire was filled out by treating physicians in the ward and used to gather information regarding the practice of pneumonia diagnosis and management. All adult patients with pneumonia were included. For inclusion, admitted patients were required to have a physician diagnosis of suspected or proven hospital acquired pneumonia (HAP), community acquired pneumonia (CAP), or aspiration pneumonia (AP). Patients with age less than 14 years and those with multiple bacterial infectious diseases, including pneumonia were excluded. Accordingly, all eligible consented patients admitted from 1 September 2016 to 30 June 2017 were approached and recruited.

Pneumonia was defined by treating physicians based on clinical examination and other diagnostic tools. All treating physicians in charge of the internal medicine wards were requested to cooperate in providing information about their respective patients diagnosed with pneumonia. In addition, the data collectors (physician interns and clinical pharmacists) had the opportunity to review patients’ charts to identify patients with pneumonia.

### Data analysis

Data entry and analyses were performed by a data clerk and the research team using IBM SPSS Statistics for Windows Version 20 (IBM Corp. Released 2012, Armonk, NY: IBM corp.). Simple descriptive statistics were used to characterize the data. Univariate analysis was performed and variables having a p-value of less than 0.25 with the outcome (either poor outcome or good outcome) were considered for further analysis. Independent predictors were then identified by multivariate logistic regression analysis. All statistical tests were 2-tailed and a p-value of less than 0.05 was considered statistically significant.

## Results

### Socio-demographic data

**Fig 1:**
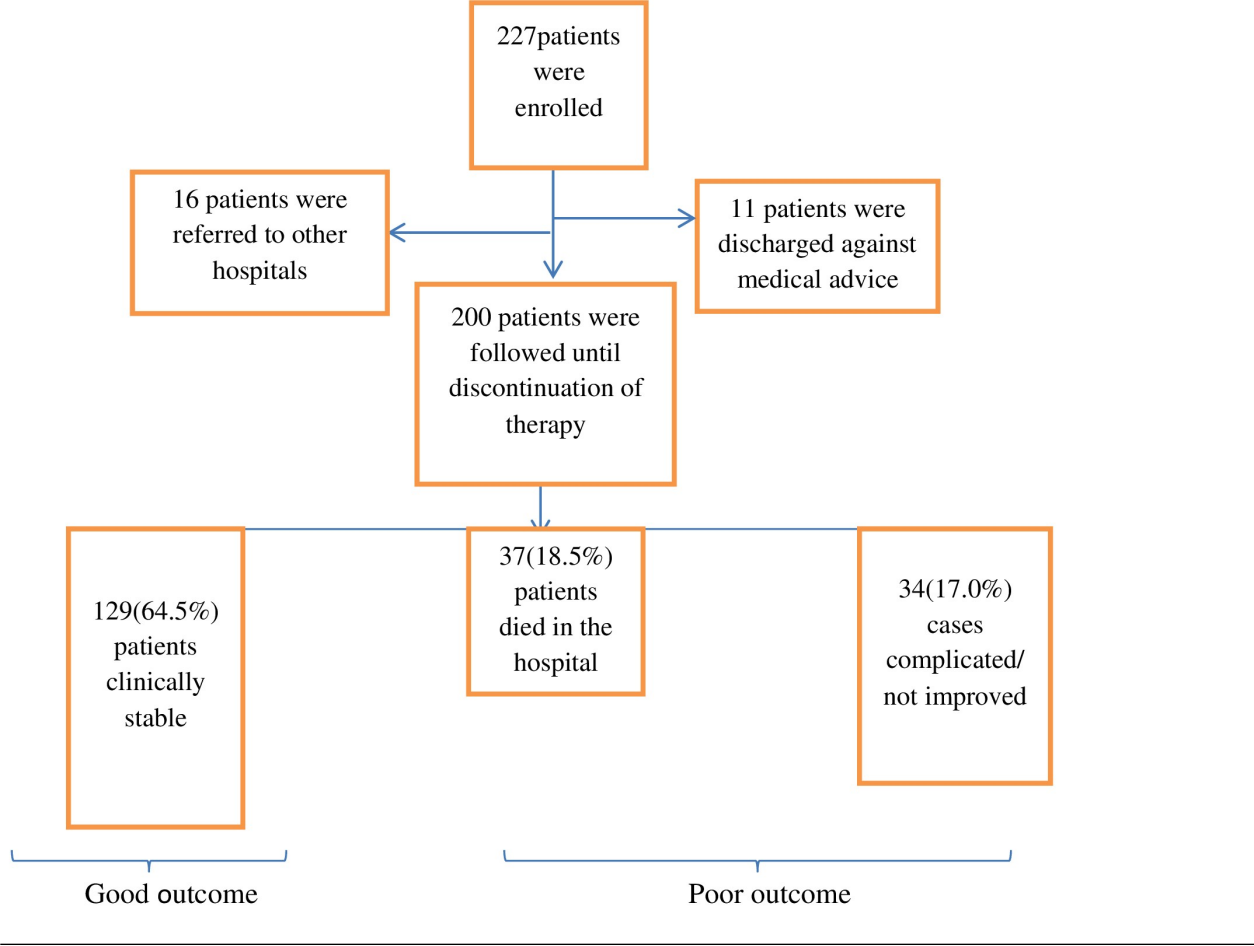
Identification and exclusion of patients included in the evaluation of the clinical outcome of adult pneumonia at Tikur Anbessa Specialized Hospital, Addis Ababa, Ethiopia, September 15, 2016- June 30, 2017 (n = 200).

A total of 227 patients who fulfilled the inclusion criteria were included in the study. However, data for 27 patients were not complete as a result of discharge against medical advice and transfer to other hospitals (Fig 1).

Socio-demographic characteristics of the study participants are described in Table 1. The data showed that more than half (104, 52.0%) of the patients were males and a majority of them (176, 88.0%) were less than 65 years of age. Mean age of the patients was 39.8 (SD 17.8) years and the large proportion (137, 68.5%) of them was referred from governmental health institutions.

**Table 1:** Socio-demographic characteristics of patients with pneumonia at Tikur Anbessa Specialized Hospital, Addis Ababa, Ethiopia, September 15, 2016- June 30, 2017 (n = 200).

Variable	Value	Number of patients (%)
Age category	<18Yrs	12(6.0)
	18-39Yrs	99(45.5)
	40-64Yrs	62(31.0)
	65-74Yrs	16(8.0)
	≥75Yrs	11(5.5)
Age (years)	Mean (SD),range	39.79(17.76),14–84
Sex of the patient	Male Female	104 (52.0) 96 (48.0)
Region from which the patient came from	Addis Ababa Oromia SNNP^#^ Amhara Others*	85 (42.5) 50 (25.0) 28 (14.0) 27 (13.5) 10 (5.0)
Referred from	Government institution Private institution Direct admission	137 (68.5) 45 (22.5) 18 (9.0)

*Afar, Ethiopia Somali, Tigray Regions

^#^ Southern nations, nationalities and people’s region

## Clinical characteristics

**Fig 2:**
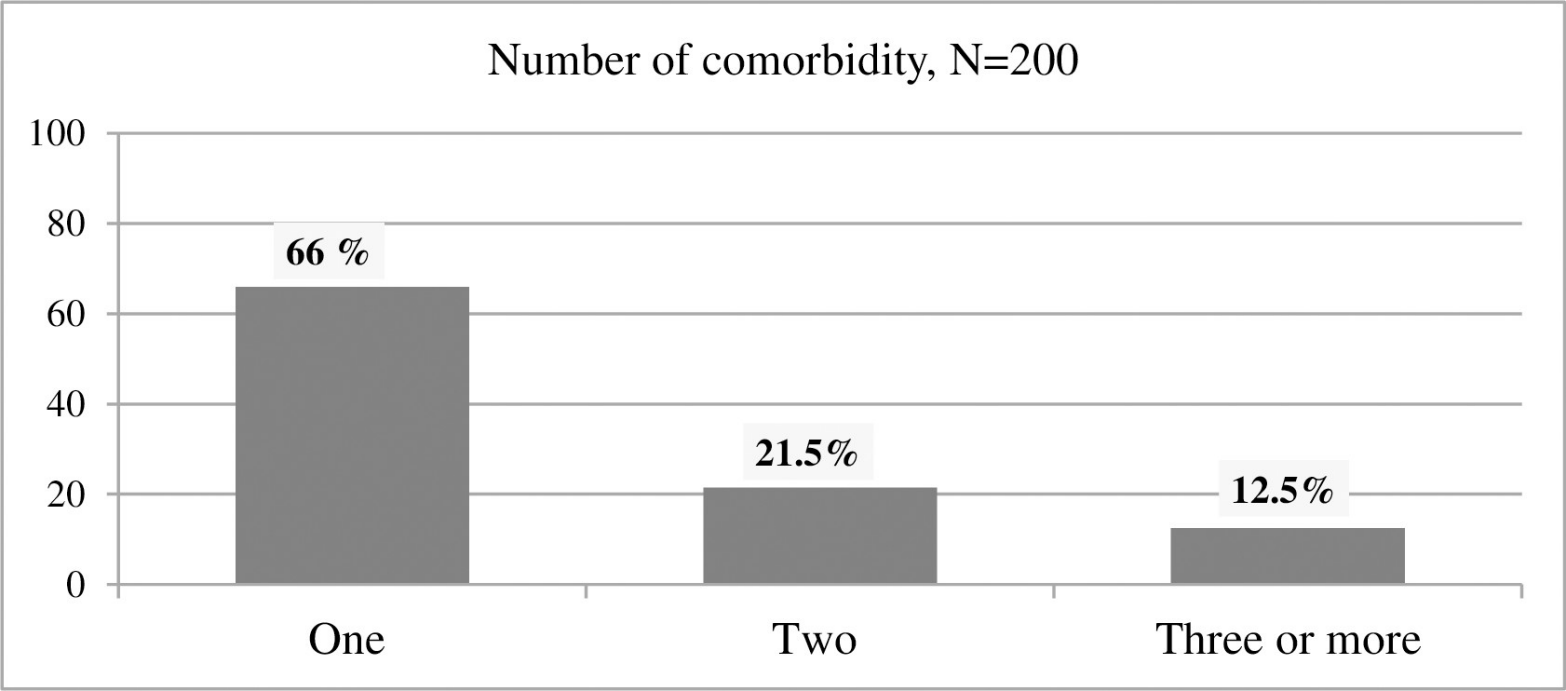
The number of co-morbid conditions of the enrolled pneumonia patients at Tikur Anbessa Specialized Hospital, Addis Ababa, Ethiopia, September 1, 2016- June 30, 2017 (n = 200).

A very small number of patients (3, 1.5%) were admitted due to pneumonia, without any other co-morbid conditions (Table 2). The most common co-morbid conditions were cancer (41.5%) and heart failure (21%) (Table 3 and Fig 2). The two most common conditions that led to admission were pneumonia with cardiovascular diseases and pneumonia with cancer (33, 16.5% for both) (Table 2). Disaggregating pneumonia into different forms revealed HAP to be the major (48.5%) form followed by CAP (41.5%) and AP alone or with HAP (10%). It is also of note that the majority (140, 70%) of patients had a recent exposure (within 90 days) to antimicrobial agents.

**Table 2:** Pneumonia patients admission diagnosis and their frequency at Tikur Anbessa Specialized Hospital, Addis Ababa, Ethiopia, September 1, 2016- June 30, 2017(n = 200).

Admission Diagnosis	N (%)
Cardiovascular Diseases + Pneumonia	33(16.5)
Cancer + Pneumonia	33(16.5)
Cancer	25(12.5)
Cardiovascular Diseases	8(4.0)
Diabetes + Cardiovascular Diseases	7(3.5)
Cardiovascular Diseases +Renal Disease + Pneumonia	7(3.5)
HIV/AIDS + Pneumonia	5(2.5)
HIV/AIDS + Cancer + Pneumonia	5(2.5)
Cancer + Renal Disease + Pneumonia	4(2.0)
Cardiovascular Diseases + Diabetes + Pneumonia	4(2.0)
Pneumonia	3(1.5)
Cancer + HIV/AIDS	2(1.0)
Others*	64(32.0)
Total	200(100)

*Different combinations of the listed diseases, systemic lupus erythematosus, Peptic ulcer disease

Visceral leishmaniosis, central nervous system disorders etc.

**Table 3:** The frequency of co-morbid conditions of the enrolled pneumonia patients at Tikur Anbessa Specialized Hospital, Addis Ababa, Ethiopia, September 1, 2016- June 30, 2017(n = 200).

Co-morbid Conditions	Yes (N, %)
Cancer	83(41.5)
Heart Failure	42 (21.0)
Chronic Pulmonary Disease	29(14.5)
Hypertension	23(11.5)
Diabetes	23(11.5)
Coronary Heart Disease	17(8.5)
Chronic Kidney Disease	14(7.0)
Stroke (new & old)	13(6.5)
Central Nervous System disorder	9(4.5)
Chronic Liver Disease	2(1.0)

## Practice of Microbiologic investigations

**Fig 3:**
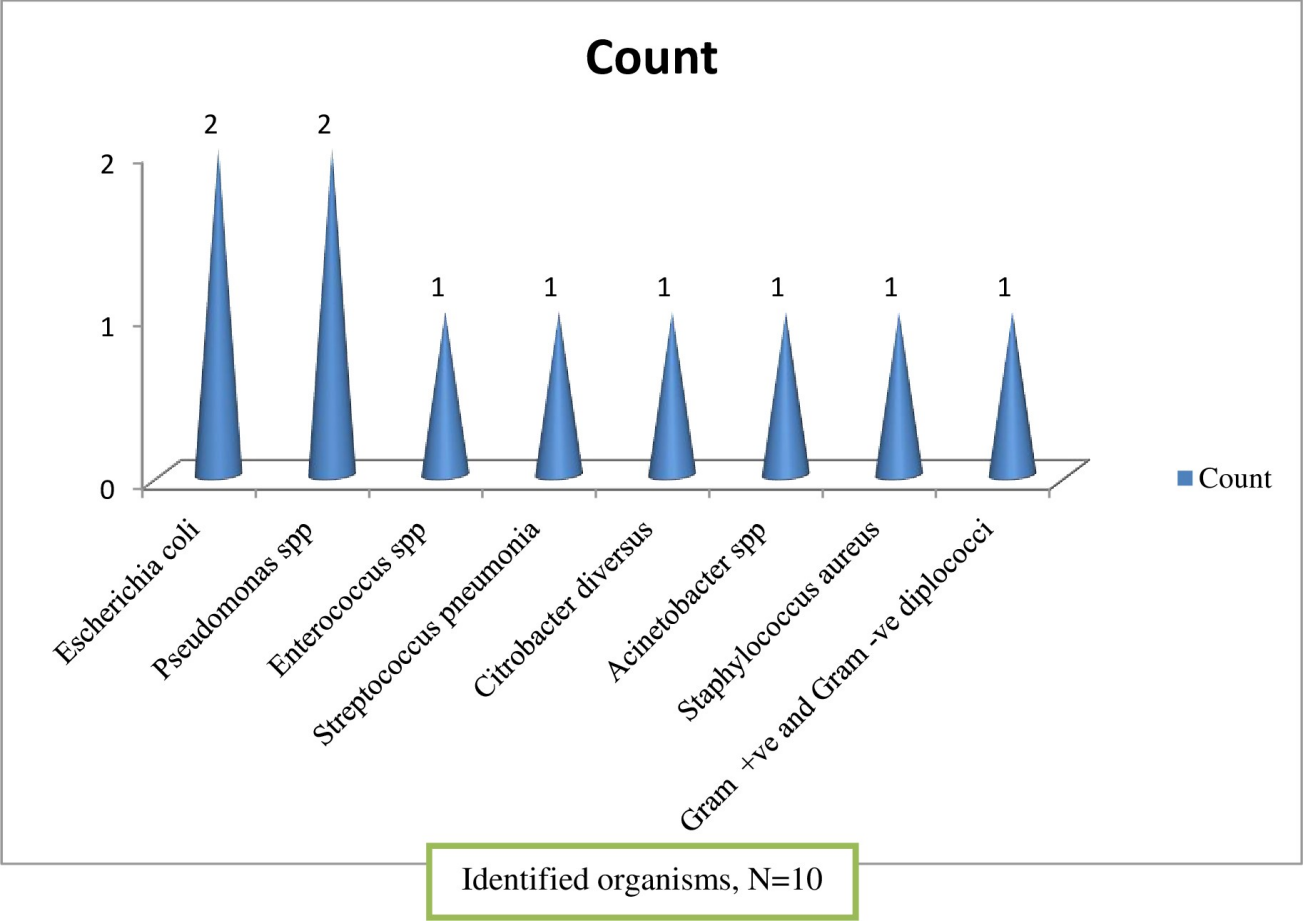
Identified organisms during the management of pneumonia at Tikur Anbessa Specialized Hospital, Addis Ababa, Ethiopia, September 1- June 30, 2017.

Microbiological tests were conducted for about a third (75, 37.5%) of the patients, out of which 60% of the samples were taken before initiation of empiric antimicrobial therapy. The most common sample taken was blood (64.0%) and the least was sputum (9.3%). The time of culture collection was found to range from 2 (2.7%) to 5 (60%) days. Bacterial growth was obtained in a relatively small (10, 13.3%) cultured microbiological samples (Fig 3), and the most commonly isolated pathogens from blood samples were *E*. *coli* and *Pseudomonas* species.

One hundred and twenty-five physicians who happened not to order microbiologic tests were asked why they did not order them, and nearly half of them said that the usual practice is treating based on clinical presentation (Table 4).

**Table 4:** Physicians’ reasons for not considering microbiological tests for the management of pneumonia patients at Tikur Anbessa Specialized Hospital, Addis Ababa, Ethiopia, September 1, 2016- June 30, 2017(n = 125).

Reasons given by physicians	N (%)
The usual practice is to treat patients based only on clinical information (signs and symptoms)	61(48.8)
The patient has already started antimicrobials	22(17.6)
No institutional guidance that recommend testing	20(16.0)
No well-equipped microbiological lab	8(6.4)
Other reasons*	14(11.2)

* Culture yields are very low and only radiologic information is sufficient to treat pneumonia

## Prescribing pattern and antimicrobial susceptibility

Almost all the treatment approaches (99.5%) were found to be empirical and no de-escalation of therapy was made even after the culture results had been obtained. The initiated antimicrobials were continued in many cases for the desired duration of therapy.

More than 30 types of antimicrobial regimens were used for management of pneumonia. The most commonly used initial antimicrobial regimens were ceftriaxone 1gm BID + azithromycin 500 mg, PO, QD, (58, 30.0%) for CAP and vancomycin 1gm BID + piperacillin/tazobactam 4.5 gm iv QID (15, 8.0%) for HAP. *E*. *coli* isolates were resistant to cephalosporins (presumed ESBL), while *Pseudomonas* isolates were susceptible to ceftazidime, aminoglycosides, quinolones, and carbapenems. By contrast, *Acinetobacter species* were resistant to all drugs tested.

It was very difficult to precisely tell the time of initiation of antimicrobials in the course of therapy. Thus, an effort was made to surmise the time of exposure to antimicrobials, as the date of diagnosis is available in the patient chart and date and time of antimicrobial administration in the medication administration sheet. Based on this information, only 127 (63.5%) patients received antimicrobials within 24 h (patients who received on the date of diagnosis). In addition, variations were observed in prescriptions with respect to selection of antimicrobials, doses, frequencies, and duration of therapy among patients with a similar diagnosis.

**Fig 4:**
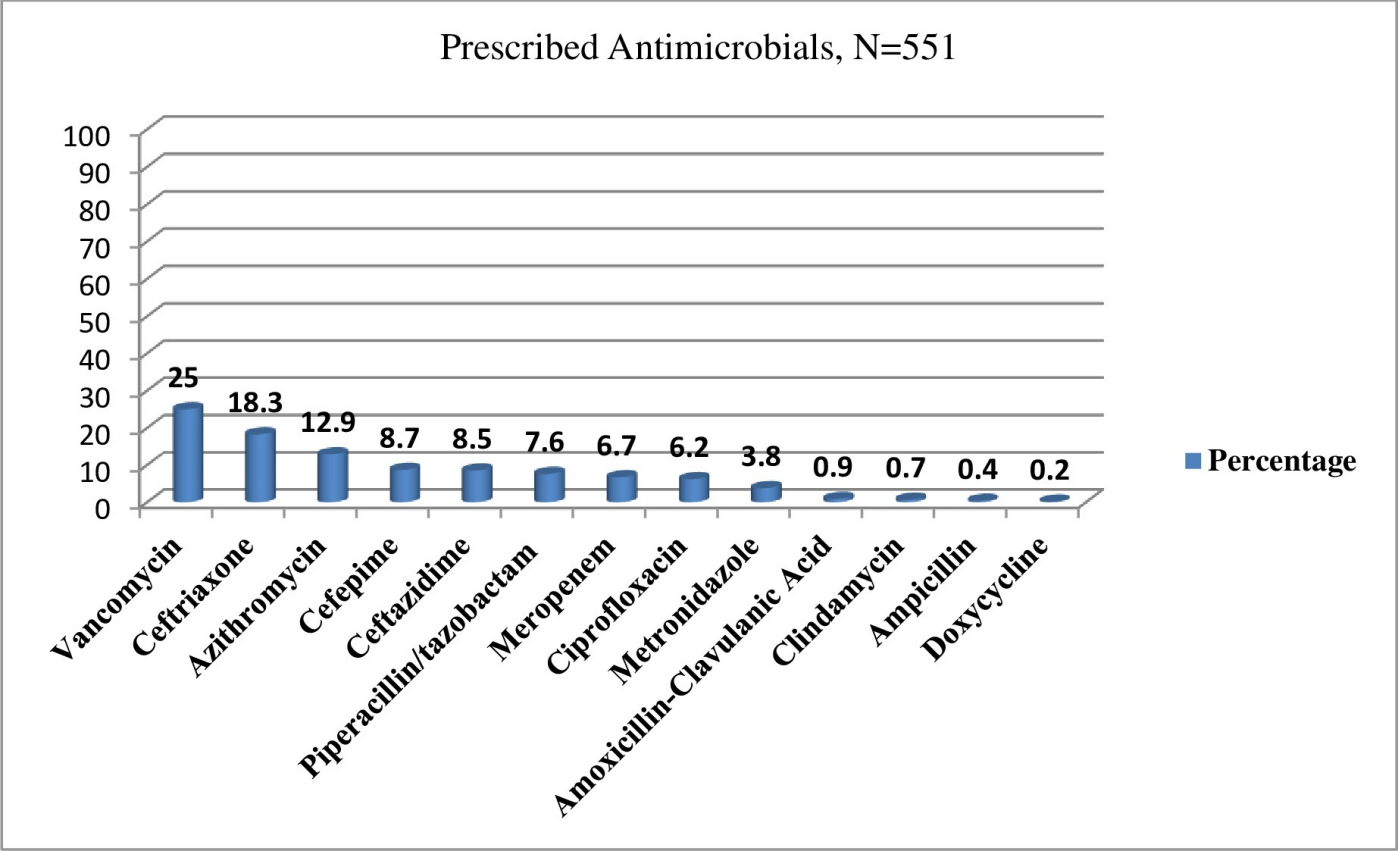
The most commonly used empiric antimicrobials for the management of pneumonia at Tikur Anbessa Specialized Hospital, Addis Ababa, Ethiopia, September 1- June 30, 2017(n = - 551).

As shown in (Fig 4), about 13 types of antimicrobials were used for the treatment of different types of pneumonia and vancomycin was the most commonly prescribed empiric antimicrobial agent (138, 69%). The average number of antimicrobials prescribed per patient were 2.76≈ 3, regardless of the type of pneumonia.

Many patients had not taken their medications as prescribed. About 60 (30.0%) of the patients missed their antimicrobial doses due to various reasons during the course of treatment (Table 5).The susceptibility data indicated that most of the identified organisms are resistant to the frequently prescribed beta-lactam antimicrobial agents (penicillins and cephalosporins).

**Table 5:** Reasons for missing antimicrobial doses during the management of pneumonia at Tikur Anbessa Specialized Hospital, Addis Ababa, Ethiopia, September 1, 2016- June 30, 2017(n = 60).

Reasons of missing doses	N (%)
Stock outs of antimicrobials in the hospital	22(36.7%)
Prescribed antimicrobials are not administered at the prescribed time.	20(33.3%)
Patients and/or their care givers didn’t provide the antimicrobials on the medical wards for timely administration	10 (16.7%)
Unknown reasons	8(13.3%)

Changing therapy was also assessed during the course of treatment. It was found that there were 83(41.5%) first time changes and the most common reason associated with the changes was poor response to the initial antimicrobials. The detailed reasons are given in Table 6. Regimens of 26 patients were also changed for a second time and 6 for a third time. Poor clinical response was the main reason for the changes.

**Table 6:** Reasons for change in initial antimicrobial regimen for the management of pneumonia at Tikur Anbessa Specialized Hospital, Addis Ababa, Ethiopia, September 1, 2016- June 30, 2017(n = 73).

Reasons for change in regimen for the first time	N (%)
Poor response	25(30.1)
Drug shortage (stock outs)	18(21.7)
Change in diagnosis	13(15.7)
Inadequate selection (broader coverage)	11(13.3)
Side effect of antimicrobials	6(9.6)
Due to discharge after being stable	2 (2.4)
Others*	8(9.6)

*Conversion to oral medications; ID physician decision

The total duration of antimicrobial therapy was 12.05(±5.09) days. Almost 70% of the patients took more than 10 days and about 35% more than 14 days.

**Fig 5:**
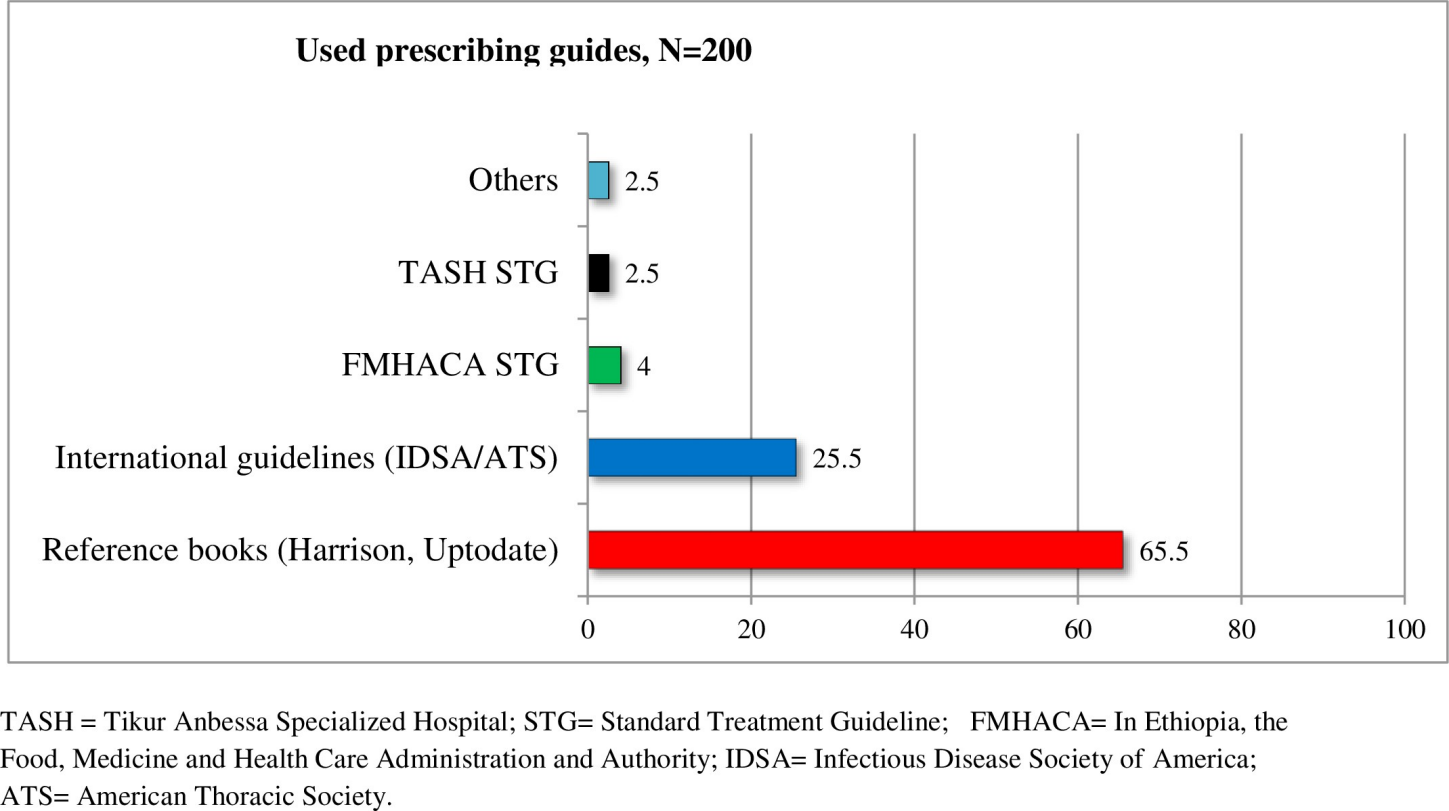
Antimicrobial prescribing guides used by physicians for the management of pneumonia at Tikur Anbessa Specialized Hospital, Addis Ababa, Ethiopia, September1- June 30, 2017(n = 200).

The most common prescribing physicians were internal medicine residents (84.5%), emergency medicine residents (12.0%), and medical interns (3.5%). Most physicians (65.5%) use reference eBooks (Harrison and UpToDate) as a guide for prescribing antimicrobials for the management of pneumonia. A small proportion (2.5%) relied on personal experience (Fig 5). Eligibility of patients for IV (intravenous) to PO (oral) conversion (based on Society for Healthcare Epidemiology of America criteria) was assessed and it was found that 67 (33.5%) patients were eligible for conversion. However, the conversion was made only for 4 (2%) patients, and even these conversions were made late. Physicians were asked about their experience with regard to IV to PO conversion and they alluded to the fact that conversion is performed mostly at the time of discharge (82, 41.8%).

## Imaging and laboratory data

Renal function tests were performed for 189 (94.5%) patients. Levels of serum BUN before the start of antimicrobials were found to be normal (3-20mg/dl) in 66 (33%) patients and 99 (49.5%) patients had >30mg/dl. Levels of serum creatinine (Scr) before initiation of antimicrobial therapy were less than or equal to 1.4mg/dl in 158 (83.6%) and greater than 1.4mg/dl in 31(16.4%) patients. After initiation of antimicrobial treatment, the proportion of patients with Scr ≤1.4mg/dl came down to 74.1% and those with >1.4 mg/dl went up to 25.9%.

Radiologic imaging, as a supplement to clinical history and physical examination in the diagnosis of pneumonia, was used only in 122(61.0%) patients. Eleven (9.0%) patients had normal radiologic findings, 65(53.3%) suggestive of pneumonia, and 46(37.7%) showed different findings, which were related to the co-morbid conditions.

## Clinical outcome

All the patients were followed up starting from the day of initiation of antimicrobial therapy until a clinical outcome was achieved and antimicrobial therapy was discontinued. The clinical outcomes were recorded immediately after completion of the treatment based on the response of the treating physician. Accordingly, 129 (64.5%) patients had a stable/improved condition, 13 (6,5%) died due to pneumonia, 24 (12%) died due to pneumonia with other co-morbidities, and 34 (17%) showed no improvement. The total in-hospital mortality was 37 (18.5%). The findings, particularly clinical stability, were not confirmed by an independent physician and might have interviewee bias (See Table 7).

**Table 7:** Cross tabulation showing the type of pneumonia and clinical outcomes at Tikur Anbessa Specialized Hospital, Addis Ababa, Ethiopia, September 1- June 30, 2017(n = 200).

Type of pneumonia	Outcome status		Total
	Good outcome N(%)	Poor outcomeN(%)	
HAP	56(28.0)	41(20.5)	97(48.5)
CAP	58(29.0)	25(12.5)	83(41.5)
Others*	15(7.5)	5(2.5)	20(10.0)
**Total**	**129(64.5)**	**71(35.5)**	**200**

*HAP + AP, HAP = Hospital Acquired Pneumonia; CAP = Community Acquired Pneumonia

AP = Aspiration Pneumonia

## Predictors of poor outcome in pneumonia patients

Since a logistic regression model requires the dependent variable to be expressed dichotomously, the clinical outcome was recoded into a good outcome (stable and improved patients) and poor outcome (in-hospital mortality and complications). Univariate binary logistic regression was used to identify independent determinants for poor outcome with a p-value of less than 0.25 and these were selected as potential predictors for further analyses. But variables like age, which happen to be clinically important, were taken as predictors of mortality even if the univariate analysis results were greater than 0.25. Multivariate logistic regression analysis was performed to assess independent predictors of poor outcome. Accordingly, patients with the following 5 characteristics demonstrated higher probability for poor outcomes: recent antimicrobial use history (p = 0.007, AOR 2.86(1.33–6.13)), cancer (p = 0.023, AOR 3.46(1.18–10.13)), recent recurrent upper respiratory tract infection (p = 0.046, AOR 3.70(1.02–13.40)), respiratory rate >24breaths/min or <12breaths/min (p = 0.013, AOR 2.45(1.21–4.95)), and serum creatinine>1.4mg/dl after the start of antimicrobial therapy (p = 0.032, AOR 2.37(1.07–5.20)) (See Table 8).

**Table 8:** Multivariate logistic regression analysis of factors associated with poor outcome among pneumonia patients who received antimicrobial therapy at Tikur Anbessa Specialized Hospital, Addis Ababa, Ethiopia, September1- June 30, 2017.

Variables		Clinical Outcome (N, %)		COR (95%CI)	AOR (95% CI)	P-value
		POC	GOC			
Sociodemographic characteristics	<18Yrs	2(2.8)	10(7.8)	1	1	
	18-39Yrs	39(54.9)	60(46.5)	3.25(0.67–15.63)	0.27(0.03–2.38)	0.240
	40-64Yrs	22(31.0)	40(31.0)	2.75(0.55–13.69)	1.17(0.26–5.11)	0.834
	65-74Yrs	4(5.6)	12(9.3)	1.67(0.25–11.07)	0.85(0.18–3.9)	0.833
	≥75Yrs	4(5.6)	7(5.4)	2.86(.41–20.14)	0.38(0.05–2.98)	0.354
Patient medical history	Recent AME					
	No	21(29.6)	59(45.7)	1	1	
	Yes	50(70.4)	70(54.3)	2.00(1.08–3.72)	2.86(1.33–6.13)	0.007
	Recent recurrent					
	URTI					
	No	62(87.3)	124(96.1)	1	1	
	Yes	9(12.7)	5(3.9)	3.6(1.16–11.19)	3.70(1.02–13.40)	0.046
Type of Pneumonia	HAP	41(57.7)	56(43.4)	1	1	
	CAP	25(35.2)	58(45.0)	0.57(0.31–1.07)	0.84(0.39–1.79)	0.660
	Others*	5(7.0)	15(11.6)	0.41(0.14–1.23)	1.13(0.31–4.07)	0.843
Type of Co-morbidity	Patients with HF	10(14.1)	30(23.3)	1	1	
	Patients with Ca	38(53.5)	44(34.1)	2.59(1.12–5.98)	3.46(1.18–10.13)	0.023
	Patients with OC	23(32.4)	55(42.6)	1.25(0.53–2.98)	1.40(0.46–4.19)	0.545
Vital Signs	Respiratory rate >24 or <12 breaths/min					
	No	28(40.0)	73(58.9)	1	1	
	Yes	42(60.0)	51(41.1)	2.15(1.18–3.90)	2.45(1.21–4.95)	0.013
Laboratory findings	Serum Cr after start of AMT					
	≤1.4mg/dl	44(66.7)	96(78.0)	1	1	
	>1.4mg/dl	22(33.3)	27(22.0)	1.77(0.91–3.46)	2.37(1.07–5.20)	0.032

*HAP + AS, AP = Aspiration pneumonia; BUN = Blood urea nitrogen; Ca = Cancer; OC = other co-morbidities; URTI = Upper respiratory tract infection

AME = Antimicrobial exposure; AMT = Antimicrobial therapy; POC = Poor clinical outcome; GOC = Good clinical outcome CI = Confidence interval, COR = Crude odds ratio; AOR = Adjusted odds ratio

## Discussion

Rational antimicrobial use is a cornerstone for the containment of antimicrobial resistance as well as good clinical and economic outcomes. However, inappropriate use of antimicrobials has a dire consequence on patients as well as the general population. This study was therefore designed to assess the practice of antimicrobial utilization and clinical outcomes in the management of adult patients with pneumonia admitted to TASH. Pneumonia was selected because it is one of the most prevalent infectious diseases in TASH.

Microbiological tests were performed only for 75 (37.5%) patients and most culture results were reported after 5 days. Studies done elsewhere, however, indicated that blood cultures were obtained either within 24 h and before the initial dose of antimicrobials (81% of patients) [18] or during admission (98% of patients) [19]. The discrepancy of these findings with the current study might be attributed to poor attention to the use of microbiological data and lack of rapid diagnostic kits. There is an ongoing study in the same setting on hospital acquired infections, which provided culture media and sensitivity discs to the hospital laboratory. Preliminary reports from this study show increased number of patients with microbiology test results suggesting that a lack of diagnostic kits is a factor explaining the present finding. Indeed, identification and detection of a pathogen susceptible or resistant to the chosen empiric antimicrobial therapy is an important outcome of microbiological studies that determines definitive therapy and infection prognosis.

The findings showed that most patients were treated without microbiological data, and this is in agreement with the treating physicians’ response (Table 4). Recommendations from published guidelines for the treatment of pneumonia advise physicians to initiate treatment with broad-spectrum antimicrobials, with appropriate de-escalation based on culture results [20]. A study [19] indicated that from 240 patients included treatment with a combination of piperacillin/tazobactam and vancomycin, and antibiotic regimens were de-escalated in 151 (63%) and 175 (73%) patients within 72 and 96 h, respectively. However, in the present study, no de-escalation of therapy was observed even after obtaining culture results. The delay in culture result collection might be a reason for the absence of de-escalation therapy, in addition to the reasons listed in the above paragraph.

Many of the clinically stable and complicated cases, who were on a combination of broad- spectrum antimicrobials, had completed the entire course. Moreover, about 30% of patients received additional broader antimicrobial agents because of poor response to initial regimens. This could be for the following reasons: i) mistrust of culture results; and ii) most physicians do not seem to consider resistance to be an important risk in the clinical context of the patient at the time of antimicrobial therapy. To be on the safe side, or not to take risk, they prescribe broad-spectrum antimicrobials [21, 22]. However, this practice might increase patients’ exposures to unnecessary antimicrobials, emergence of resistance, and increased health care costs [23, 24]. In general, the data suggests that the use of microbiological data is very limited not only in the management of pneumonia but also in the management of other infectious diseases. Such practice at a tertiary care level hospital is worrisome.

Almost all treatment approaches were empirical (199, 99.5%) in the current study. Empiric antimicrobial therapy is generally categorized as appropriate (adequate) or inappropriate (inadequate) based on microbiological culture and susceptibility findings. Empiric therapeutic regimens are considered appropriate if the identified microorganism is susceptible to at least one of the antimicrobial agents [25, 26]. The yield of culture-positive results from all types of samples using the traditional microbiology panel is usually low [27]. This may be due to prior antimicrobial exposure before sample collection [28], sample type tested, and the diagnostic tool used for patient evaluation[29]. In this study, however, only four out of ten patients who received empiric antimicrobial regimens had culture and susceptibility testing results with the appropriate coverage. Other studies have shown that appropriate empiric antimicrobial therapy is associated with decreased mortality of patients with many different types of infections. Nevertheless, absence of culture and susceptibility data could also have negative effects on patient outcomes as well as on the economy [11, 12, 30].

More than 30 types of antimicrobial regimens were used in the present study. Particularly, empiric use of very costly and lifesaving antimicrobial agents like vancomycin, meropenem and third and fourth generation cephalosporins (ceftriaxone, ceftazidime, and cefepime) was found to be common in this hospital. It was noted that there were a number of factors that influence antimicrobial selection. Firstly, the absence of a standardized hospital-specific protocol encourages physicians to use an antimicrobial agent of their own selection. Secondly, frequent stock-outs of most of the antimicrobials in the hospital could lead the treating physician to prescribe the available antimicrobial agents in the inpatient pharmacies. Hence, an appropriate strategy should be put in place in the selection of empiric antimicrobial agents so as to minimize their overuse and/or unnecessary use. This should be coupled to instituting mechanisms to ensure continuous supply of the needed antimicrobial agents.

Out of 551 prescriptions, vancomycin was the most commonly used (138, 25%) empiric antimicrobial agent for pneumonia management at TASH. This finding is similar to a study done by Nak-Hyun Kim *et al*,[31] on empiric use of vancomycin for most frequent clinical reasons of pneumonia. The study revealed that the empiric use of vancomycin was discontinued within 96 h in 39.0% of prescriptions (187/480 prescriptions), but used continuously for ≥96 h in 61.0% (293/480) prescriptions [31]. Antimicrobial de-escalation is a strategy for proper antimicrobial utilization to balance empiric use and reduce the emergence of resistance. However, this is not practiced within the hospital. In addition, missed doses were observed in 60 (30.0%) of the patients, which, in turn, might have resulted from the absence of a clinical pharmacist assigned to work in the wards who could provide consultation on antimicrobial utilization and pharmaceutical care services to patients. All of these factors could contribute to the rapid development of antimicrobial resistance.

The use of local guidelines in the management of pneumonia was minimal in this study. The reason could be the absence of local standard treatment guidelines tailored for a tertiary care hospital. The guidelines developed for zonal hospitals were prepared without local antibiogram data, forcing physicians to depend mainly on reference books and international guidelines. However, different guidelines and literature are published at different times and in many cases, their recommendations are not the same [32, 33], leading to the diverse use of antimicrobial agents in the management of patients with similar pneumonia diagnoses. Moreover, the indicated reference books and international guidelines are prepared based on their own country antimicrobial resistance patterns and most of them are intended for educational purposes. Thus, the guidelines recommend having institution-specific guidelines developed based on institutional antibiograms [20].

Out of 551 antimicrobial courses from 67 eligible patients for possible IV to PO conversions, only 4 were converted after several doses of IV antimicrobials. A similar pattern was reported in a Lebanese hospital [34], where only a small proportion of treatment courses were switched. This could be attributed to limited awareness of the advantages of IV to PO conversion and physicians’ attitude towards effectiveness of PO antimicrobials. This was reflected in their response to the question regarding the very limited practice of IV to PO conversion. About 82 (41.8%) of the treating physicians said that administration of IV antimicrobials for hospitalized patients is a usual practice in this hospital and conversion is only made at the time of discharge. This thought is also shared by other studies, in which about 47% of the physicians stated that patients should receive a standard duration of IV antibiotics [35]. However, studies clearly demonstrate that the average expense for antimicrobials and the length of stay of patients could be reduced from early IV to PO conversion [36]. Antimicrobial agents cost is about 25 to 40% of the total medication budget in hospitals in the United States [37, 38]. This is believed to be much higher in the Ethiopian context, where the prevalence of infectious diseases is very high [39]. Thus, considerations of possible conversions are crucial in a resource-limited country like Ethiopia.

In the current study, the overall in-hospital mortality was 37 (18.5%). In addition, the disease worsened in about 34 (17%) patients, though two or more combinations of antimicrobials were administered for more than 10 days. This could emanate from misdiagnosis or infection with a resistant pathogen (s). The former could possibly be attributed to the absence of microbiological and radiologic examinations and the latter due to past exposure to antimicrobials prior to admission to TASH. The in-hospital mortality rate is very high (18.5%) compared to other similar studies[18, 40], suggesting that the quality of care of patients with infectious diseases is minimal at TASH. Absence of a sufficient number of infectious disease specialists, infectious disease-trained clinical pharmacists, hospital-specific antimicrobial treatment protocols, continuous supply of antimicrobials and better microbiological laboratory services could be cited as possible reasons for the poor quality of care.

Identification of risk factors for poor outcome is an important strategy during infectious disease management. Because it provides a clue for better care according to the number of risk factors associated with patients. Hence, this study attempted to assess the possible predictors of poor outcome in patients with pneumonia. Accordingly, around five risk factors were identified and these risk factors are in line with other studies done across the globe.

Recent antimicrobial use history (p = 0.007) was one of the predictors of poor clinical outcome. Many studies have reported that prior antimicrobial drug exposure is associated with colonization and infection by resistant pathogens [23, 24, 41] as well as increased mortality [42]. As patients are referred from lower-tier health institutions, the likelihood of taking antimicrobials prior to coming to TASH is high. A significant association also was observed between cancer and poor outcome (p = 0.023) in patients with pneumonia. This is also consistent with other studies [43], where mortality was shown to be higher in pneumonia patients with than without cancer. Several other studies also demonstrated that the mortality of cancer patients with lower respiratory tract infections is very high[44, 45]. Therefore, pneumonia patients with malignancies would require careful workup and frequent evaluation of antimicrobial therapy.

History of prior recurrent upper respiratory tract infection (URTI) is also found to be predictor of poor outcome (p = 0.046). URTI is a risk factor for pneumonia especially in immunocompromised patients [46]. Many of the patients included in this study were immunocompromised (44%) and might have experienced recurrent bacterial infections. Hence, consideration of past medical history in antimicrobial therapy is crucial to decrease the risk of morbidity as well as mortality.

High level of Scr after the start of antimicrobial therapy was associated with increased mortality in patients with pneumonia (p = 0.026). Many of the patients included in this study had comorbidities and most of them were taking nephrotoxic drugs such as vancomycin alone or in combination with ceftazidime, piperacillin-tazobactam, cefepime, or meropenem that could result in acute kidney injury. Collectively, damage to the kidney due to existing comorbidities, antimicrobials and the infection itself could have resulted in an increased risk of mortality in the study participants. Studies have also shown that elevated serum creatinine in CAP is associated with increased 30 day mortality [47].

Patients with respiratory rate (RR) >24breaths/minute or <12breaths/minute were associated with increased mortality (p = 0.013) and this is consistent with other studies conducted elsewhere[48].

## Strengths and limitations of the study

The strengths of this study include longer follow up and documentation of clinical and laboratory variables at admission for baseline as well as for follow up on each consecutive days after initiation of treatment.

The primary limitation of this study was poor documentation: First, the treating physicians did not document all the necessary information at the time of admission and initiation of antimicrobial therapy. Second, nurses did not properly document the administered antimicrobials in the medication administration sheet. Vital signs also were not timely and properly recorded.

Important laboratory tests such as blood glucose level and serum albumin were not done for most of the patients.

## Conclusion

Antimicrobial use in TASH is complicated by different service-related factors and physician attitudes. Antimicrobials are used without sufficient evidence of indication and microbiological and radiological findings. The practice also is not supported with relevant local guidelines and no multidisciplinary approach was apparent in the management of infectious diseases. [How about: It is likely that these factors contributed to higher rates of mortality (18.5%) when compared with similar studies in other countries ] As a result, there were higher rates of mortality (18.5%) when compared with similar studies in other countries. In general, to improve proper antimicrobial utilization and patient clinical outcomes, the hospital requires a coordinated intervention from all concerned bodies, including a functional antimicrobial stewardship program as soon as possible.

## Supporting information

S1 File Data collection format. (DOCX)

S2 File SPSS Regression output. (DOCX)

S3 File Raw data for regression output. (DOCX)

## Abbreviations

AAU: Addis Ababa University
BID: Bis In Die (Twice a Day)
CAP: Community Acquired Pneumonia
CDC: Center for Disease Control and Prevention
CDDEP: Center for Disease Dynamics, Economics & Policy
CHS: College of Health Science
DACA: Drug Administration and Control Authority
FMHACA: Ethiopian Food, Medicine & Healthcare Administration & Control Authority
FDA: United States Food and Drug Administration
FMOH: Federal Ministry of Health
HAP: Hospital Acquired Pneumonia
ICU: Intensive Care Unit
NGO: Nongovernmental Organization
PFSA: Pharmaceutical Fund and Supply Agency
QD: Quaque Die (Once Per Day)
QID: Quater In Die (Four Times a Day)
SHEA: Society for Healthcare Epidemiology of America
SOM: School of Medicine
SOP: School of Pharmacy
STG: Standard Treatment Guideline
TASH: Tikur Anbessa Specialized Hospital
TID: Ter In Die (Three Times a Day)
WHO: World Health Organization
