# Supplementary material for: Evaluation of current practice of antimicrobial use and clinical outcome of patients with pneumonia at a tertiary care hospital in Ethiopia: A prospective observational study
Source: PLoS One. 2020 Jan 30;15(1):e0227736. doi: 10.1371/journal.pone.0227736 (PMC6992215; doi:10.1371/journal.pone.0227736)
Supplement: S1 File — (DOCX) [file pone.0227736.s001.docx]

በአዲስ አበባ ዩኒቨርሲቲ

ጤና ሳይንስ ኮሌጅ

የፋርማሲ ትምህርት ቤት

ጥቁር አንበሳ ስፔሻላይዝድ ሆስፒታል ውስጥ በመታከም ላይ በሚገኙ የሳንባ ምች (ኒሞኒያ) ህሙማን ስለህክምና አሰጣጥ ሁኔታ መረጃ ለመሰብሰብ የተዘጋጀ መጠይቅ ነው፡፡

**መለያ ቁጥር/card no.:**____________________

ተሳታፊዎች በጥናቱ ለመሳተፍ ፈቃደኝነታቸዉን የሚገልፁበት ቅጽ

ጤና ይስጥልኝ እኔ __ቴዎድሮሰ ፈንታ__ እባላለሁ በአሁኑ ወቅት በአዲስ አበባ ዩኒቨርሲቲ፣ ፋርማሲ ትምህርት ቤት በፋርማኮሎጅና ክሊኒካል ፋርማሲ ትምህርት ክፍል የሁለተኛ ድግሪ ተማሪ ነኝ፡፡ በመሰራት ላይ ያለው ጥናት የሳንባ ምች (ኒሞኒያ) ህሙማን ህክምና አሰጣጥ ሁኔታን ለመገምገም እና የሕክምና አሰጣጡን ለማሻሻል ኢላማ አድርጎ የተነሳ ጥናትና ምርምር ነው፡፡ መረጃ መሰብሰቢያ ቅጹም ይህንኑ አላማ አድርጎ የተዘጋጀ ነው፡፡ በመሆኑም ከእርስዎ ፡ ከእርስዎ ካርድ ላይ እና ከሐኪምዎ መረጃ ለመውሰድ እንፈልጋለን፡፡ በዚህ ጥናት ውስጥ የርስዎ ተሳታፊነት ሙሉ በሙሉ በርስዎ ፈቃደኝነት ላይ የተመሰረተ ነዉ፤ በዚህ ጥናት ውስጥ መሳተፍዎም ሆነ ላለመሳተፍ መወሰንዎ በሆስፒታሉ ውስጥ በሚያገኙት አገልግሎት ላይ ምንም አይነት ተጽእኖ የማይኖረው ሲሆን ተሳትፎውን በማንኛውም ሰአት ማቋረጥ ይችላሉ፡፡ ለጥናቱ የሚያስፈልጉት መረጃዎች ሙሉ በሙሉ በምስጢር የሚጠበቁ ሲሆን የርስዎም ስም በማንኛዉም መልኩ በጥናቱ ውስጥ አይገለጽም፤ እንዲሁም የሚሰበሰቡት መረጃዎች ከርስዎ ማንነት ጋር በማንኛውም መልኩ አይያያዝም፡፡

በጥናቱ ለመሳተፍ ፈቃደኛ ነዎት?

አዎ አይደለሁም

ፈቃደኛ መሆናቸውን ካረጋገጡ መረጃ መሰብሰብ ይጀምሩ

ፈቃደኛ ካልሆኑ ወደ ሌላ ተገልጋይ ይሸጋገሩ

**Data collection format**

1. Demographic characteristics:
   1. Age:____________
   2. Sex:____________
   3. Region: _____________
   4. Card No:______________
   5. Referred from: _________
   6. Date of admission:_____________
2. Past medical history:___________________________
3. Past surgical history:_______________________________
4. Does the patient have recent antimicrobial medication use history? Yes No
5. Admission diagnosis :_________________________________________________________
6. Date of pneumonia diagnosis_________________________
7. Type of pneumonia:-
   - 1. Hospital acquired pneumonia (HAP)
     2. Community acquired pneumonia (CAP)
     3. Health care associated pneumonia (HCAP)
     4. Aspiration Pneumonia (AP)
     5. Other specify:______________________
8. Factors that predispose pneumonia (You can mark “√” one or more risk factors)
   1. Cigarette smoking
   2. Upper respiratory tract infections
   3. Alcohol
   4. Corticosteroid therapy
   5. Old age (age >65)
   6. Recent influenza infection
   7. Pre-existing lung disease
   8. Others ,Specify________________
9. Change in diagnosis (if any):___________________
   1. Date of change: ______________
   2. Reason of change (Ask the physician in charge):
      1. Additional Investigation data obtained
      2. New clinical sign and symptom observed
      3. Senior consultation (specify) _____________________
      4. Other (specify)________________
10. Is there microbiologic test (gram stain and/or culture and sensitivity)?
    1. Yes
    2. No
11. Date of sampling for the microbiologic test___________________
12. If yes to question 10, microbiologic study results:
    1. Source:_________________ gram stain:________________________
    2. Causative pathogen (s):_______________
    3. Susceptibility data:________________
    4. Time of culture collection:__________
13. If no to question number 10, what was the reason (Ask the physician in charge)?
    1. No institutional guidance that recommend testing
    2. No well equipped microbiology lab
    3. This is the usual practice
    4. Other reasons, specify____________
14. Initial antimicrobial therapy? Empiric definitive
15. Initial Empiric/definitive Antibiotic therapy:
    1. Name of antimicrobial (s), dose, route, frequency:_____________________________________________________________________________________________________________________________________
    2. Time and date of initiation:____________________________________________
    3. Duration:__________________________________________________________
    4. Any missed doses and reason__________________________________________
16. Prescribing physician (empiric or definitive):
    1. infectious disease physician
    2. infectious disease fellow
    3. internal medicine resident
    4. internist
    5. emergency medicine resident
    6. emergency medicine specialist
    7. other specify
17. Is there any medication use consultation with a clinical pharmacist?
    1. Yes
    2. No
18. If the response is No? What was the reason?
    1. Not considering their role in patient management
    2. Absence of assigned clinical pharmacist in the ward
    3. The clinical pharmacist is not interested in patient management
    4. Other specify ………………..
19. Which guideline is used to prescribe the above (q. 15) medication (s) to the patient?
    1. FMHACA ‘s STG
    2. Institutional guideline/protocol (TASH)
    3. Other international guideline (s) (specify):_____________
    4. Reference Book (s) (specify)________________________
    5. Other (specify)___________________________________
20. Is there a change in antimicrobial agent (s) or regimen?
    1. Yes
    2. No
21. If yes to the above question, mention the following information
    1. **1^st^ time change**: Name of antimicrobial (s), dose, route, frequency:____________________
    2. Time and date of change: ___________________
    3. Duration:_______________________
    4. Any missed doses and reason:________________________________
    5. **2^nd^ time change**: Name of antimicrobial (s), dose, route, frequency:____________________
    6. Time and date of change: ___________________
    7. Duration:_______________________
    8. Any missed doses and reason:________________________________
    9. **3^rd^ time change**: Name of antimicrobial (s), dose, route, frequency:____________________
    10. Time and date of change: ___________________
    11. Duration:_______________________
    12. Any missed doses and reason:________________________________
22. Reason for change:
    1. Poor response
    2. Inadequate selection
    3. Side effect of antimicrobials
    4. Drug shortage
    5. Not defined
    6. Change in diagnosis
    7. Other specify:_____________________
23. Prescribing physician (the physician that changes the regimen):
    1. Infectious disease physician
    2. Infectious disease resident
    3. Internist
    4. Internal medicine resident
    5. other (specify)_______
24. Route of drug administration changes:

- **Patient Eligibility Criteria and incidence of conversion**
- Criteria for patient eligibility
  - 1. **Inclusion criteria for IV to PO therapy conversion**
- Intravenous antimicrobial for ˃ 24 hrs
- Clinical improvement (Temp. < 37.8^0^C, O_2_ saturation >92%, stable blood pressure, Pulse rate <100 beats.min^-1^, respiratory rate, <25 breaths.min^-1^ )
- Afebrile for ˃24 hours (core temperature <38^o^C)
- Oral administration of fluids is feasible
- Oral administration of tablets is feasible
  - 1. **Exclusion criteria for IV to PO therapy conversion**
- Oral routes compromised (vomiting ¸nil by mouth, severe diarrhea , swallowing disorder, unconscious )
- Quinolone Exclusion for those receiving continuous enteral feeds
- An appropriate oral medication is not available
- Patients develop sepsis after pneumonia diagnosis (ie.2 or more the following: temp > 38 or <36_­­_^o^C,heart rate > 90bpm,respiratory rate >20 breath/minute, WCC > 12 x 10^3^/micL or < 4 x 10^3^/micL) / deteriorating clinical condition (32) .
- Patients develop febrile neutropenia after pneumonia diagnosis (ANC less than 1 x 10^9^ /L)
- Patients develop Serious deep seated infection after pneumonia diagnosis that requires IV therapy as a co-morbid (e.g. meningitis, endocarditis, infection of a prosthetic device.
  1. Is the patient eligible based on the inclusion/exclusion criteria?

Yes No

- 1. Is there IV-to-PO route conversion?

Yes No

- 1. Instead of IV drugs, is oral medication started without fulfilling of eligibility criteria?

Yes No

- 1. If the answer is “Yes”, Reasons…….
     1. Absence of first line IV medication
     2. Patient doesn’t tolerated IV routes
     3. Others , specify _____________________________________
  2. Which oral medication is used because of absence of first line IV medication ______________________________________________
  3. Are there any other oral drug prescriptions prescribed for and received by patients while on IV antibiotic therapy. Yes No
  4. If your answer yes, list the drugs and its indication__________________________

_________________________________________________________________

- 1. Was conversion made after fulfilling of eligibility criteria?
     1. Yes
     2. No conversion
  2. If the answer to query no. 24.2 is “Yes”. Which to which?

Intravenous (IV) Oral (PO)

Start date _________________ Conversion date _______________

Start time _________________ Conversion time_______________

Name1.____________________ Name1.______________________

2.___________________ 2.______________________

3. ____________________ 3.______________________

Dose_____________________ Dose _____________________

Frequency_________________ Frequency ___________________

Duration__________________ Duration ____________________

- 1. At what time was conversion made?
- Converted within 24 hr after fulfilling eligibility criteria
- Converted in between 24-48 hr after fulfilling eligibility criteria
- Converted in between 48-72 hr after fulfilling eligibility criteria
- Converted after 72 hr of fulfilling eligibility criteria

Number of Days, Date & Time, specified;____________________

- IV stopped lately after fulfilling eligibility criteria

Number of Days after clinical stability, Date &Time specified;_________________

- Converted upon discharge

Number of Days, Date &Time specified___________________________________

- IV stopped at the point that switching becomes possible

Number of days__________________________________

- IV to PO converted without fulfilling eligibility criteria

Note. If IV therapy was stopped on the day clinical stability is achieved and no oral therapy was initiated, the patients are categorizing as ‘IV stopped at point that switching become possible’.

- 1. Follow-up patient status after IV to PO conversion (at least 72 hours).
     1. Continued PO until discharged/clinical stability. Yes No
     2. Converted to other PO agents :- After omission of the first PO medication

Additional PO medication

For additional PO medication: - Name, dose, frequency ____________________

Reason/Indication _____________________Duration ___________Cost_­­­­­­______

Start Date & Time___________________ End Date & Time________________

- - 1. Converted back to IV agents to previous one OR other agent

Name, dose, frequency ___________________________________________

Reason/Indication_______________________________________________

Duration_______________________ Cost____________________________

- 1. If the answer is **“No”** for question number **24.8** (No conversion made after fulfilling of eligibility criteria) and continuation of IV therapy on day 3 or more than 3 days of treatment. Why? (Ask the treating physician)__________________________________________________________

________________________________________________________________

1. Clinical Outcome:
   1. Clinical cure/stability

- 1. Mortality Due To Pneumonia

- 1. All- cause mortality

- 1. Other (specify)___________________

1. Clinical observed: Cough Sputum production Fever

1. Vital signs:

| V/s start from pneumo dx | **Date/Time** | | | | | | | | | | | | |
| --- | --- | --- | --- | --- | --- | --- | --- | --- | --- | --- | --- | --- | --- |
|  | Normal range |  |  |  |  |  |  |  |  |  |  |  |  |
| T(C)ͦ | 36.5-37.2 |  |  |  |  |  |  |  |  |  |  |  |  |
| BP(mmHg) | <120/80 |  |  |  |  |  |  |  |  |  |  |  |  |
| PR (per min) | 60-100bts/min |  |  |  |  |  |  |  |  |  |  |  |  |
| RR (per min) | 12-24bre/min |  |  |  |  |  |  |  |  |  |  |  |  |
| SaO_2_ on atm. | >92% |  |  |  |  |  |  |  |  |  |  |  |  |
| SaO_2_ on oxy. | >95% |  |  |  |  |  |  |  |  |  |  |  |  |

- 1. Lab findings: start from pneumonia dx and last result when medication is stopped

| **Tests** | |  | **Results** | | | | | |
| --- | --- | --- | --- | --- | --- | --- | --- | --- |
| **parameters** | | **Ranges** | **Initial value** | **End of Rx** | | **Remark** | | |
|  | |  |  |  |  |  |  |  |
| **CBC/FBC** | WBC/TLC |  |  |  | |  | |  |
|  | Neutrophils% |  |  |  | |  | |  |
|  | Hb test |  |  |  | |  | |  |
|  | PCV/Hct |  |  |  | |  | |  |
|  |  |  |  |  | |  | |  |
|  | ANC |  |  |  | |  | |  |
| **LFTs** | Bilirubin total |  |  |  | |  | |  |
|  | Direct/conj. |  |  |  | |  | |  |
|  | ALT/SGPT |  |  |  | |  | |  |
|  | AST/SGOT |  |  |  | |  | |  |
|  | ALP |  |  |  | |  | |  |
|  | LDH |  |  |  | |  | |  |
|  | Albumin |  |  |  | |  | |  |
|  | Total Serum Prot. |  |  |  | |  | |  |
| **RFRs** | BUN |  |  |  | |  | |  |
|  | Cr_s_ |  |  |  | |  | |  |
|  | UA |  |  |  | |  | |  |
| **BG & HbAIc & OTHER TESTS** | RBS |  |  |  | |  | |  |
|  | FBS |  |  |  | |  | |  |
|  | HbAIc |  |  |  | |  | |  |
|  |  |  |  |  | |  | |  |
|  |  |  |  |  | |  | |  |
|  |  |  |  |  | |  | |  |
|  |  |  |  |  | |  | |  |
| **Radiology** |  | **date** |  | | | | | |
|  | **CXR** |  |  | | | | | |
|  | **Chest CT** |  |  | | | | | |
| **Other tests** |  |  |  |  |  | |  | |

**Exclusion criteria**

- Patients having bacterial infections other than pneumonia
- Age less than 14 years
