# Supplementary material for: Evaluation of current practice of antimicrobial use and clinical outcome of patients with pneumonia at a tertiary care hospital in Ethiopia: A prospective observational study
Source: PLoS One. 2020 Jan 30;15(1):e0227736. doi: 10.1371/journal.pone.0227736 (PMC6992215; doi:10.1371/journal.pone.0227736)
Supplement: S2 File — (DOCX) [file pone.0227736.s002.docx]

**Logistic Regression**

[DataSet1] C:\Users\Theodros\Desktop\Research papers\SPSS final\The refined crude data after Plos.sav

| **Case Processing Summary** | | | |
| --- | --- | --- | --- |
| Unweighted Cases^a^ | | N | Percent |
| Selected Cases | Included in Analysis | 184 | 92.0 |
|  | Missing Cases | 16 | 8.0 |
|  | Total | 200 | 100.0 |
| Unselected Cases | | 0 | .0 |
| Total | | 200 | 100.0 |
| a. If weight is in effect, see classification table for the total number of cases. | | | |

| **Dependent Variable Encoding** | |
| --- | --- |
| Original Value | Internal Value |
| good outcome | 0 |
| poor outcome | 1 |

| **Categorical Variables Codings** | | | | | | |
| --- | --- | --- | --- | --- | --- | --- |
|  | | Frequency | Parameter coding | | | |
|  |  |  | (1) | (2) | (3) | (4) |
| Age recoded | <18Yrs | 11 | .000 | .000 | .000 | .000 |
|  | 18-39Yrs | 94 | 1.000 | .000 | .000 | .000 |
|  | 40-64Yrs | 56 | .000 | 1.000 | .000 | .000 |
|  | 65-74Yrs | 12 | .000 | .000 | 1.000 | .000 |
|  | >=75Yrs | 11 | .000 | .000 | .000 | 1.000 |
| type of pneumonia | HAP | 91 | .000 | .000 |  |  |
|  | CAP | 75 | 1.000 | .000 |  |  |
|  | others | 18 | .000 | 1.000 |  |  |
| Types of co-morbidity | Pneumonia with heart failure | 35 | .000 | .000 |  |  |
|  | Pneumonia with cancer | 76 | 1.000 | .000 |  |  |
|  | Pneumonia without of heart failure & cancer | 73 | .000 | 1.000 |  |  |
| creatinine after the start of Rx | Less than or equal to 1.3mg/dl | 137 | .000 |  |  |  |
|  | greater than 1.4mg/dl | 47 | 1.000 |  |  |  |
| Factors that predispose pneumonia,upper respiratory tract infections | yes | 13 | 1.000 |  |  |  |
|  | no | 171 | .000 |  |  |  |
| respiratory rate<12 or > 24' | yes | 88 | 1.000 |  |  |  |
|  | No | 96 | .000 |  |  |  |
| recent antimicrobial use (within 90) | yes | 112 | 1.000 |  |  |  |
|  | no | 72 | .000 |  |  |  |

**Block 0: Beginning Block**

| **Classification Table^a,b^** | | | | | |
| --- | --- | --- | --- | --- | --- |
|  | Observed | | Predicted | | |
|  |  |  | outcome status | | Percentage Correct |
|  |  |  | good outcome | poor outcome |  |
| Step 0 | outcome status | good outcome | 119 | 0 | 100.0 |
|  |  | poor outcome | 65 | 0 | .0 |
|  | Overall Percentage | |  |  | 64.7 |
| a. Constant is included in the model. | | | | | |
| b. The cut value is .500 | | | | | |

| **Variables in the Equation** | | | | | | | |
| --- | --- | --- | --- | --- | --- | --- | --- |
|  | | B | S.E. | Wald | df | Sig. | Exp(B) |
| Step 0 | Constant | -.605 | .154 | 15.374 | 1 | .000 | .546 |

| **Variables not in the Equation** | | | | | |
| --- | --- | --- | --- | --- | --- |
|  | | | Score | df | Sig. |
| Step 0 | Variables | recentantimicrobialusehistory(1) | 8.890 | 1 | .003 |
|  |  | Typescomorbidity | 7.892 | 2 | .019 |
|  |  | Typescomorbidity(1) | 6.521 | 1 | .011 |
|  |  | Typescomorbidity(2) | .773 | 1 | .379 |
|  |  | URTIs(1) | 4.207 | 1 | .040 |
|  |  | respiratoryrate(1) | 7.573 | 1 | .006 |
|  |  | typeofpneumonia | 2.295 | 2 | .318 |
|  |  | typeofpneumonia(1) | 1.203 | 1 | .273 |
|  |  | typeofpneumonia(2) | .498 | 1 | .481 |
|  |  | Agerecoded | 2.698 | 4 | .610 |
|  |  | Agerecoded(1) | 1.370 | 1 | .242 |
|  |  | Agerecoded(2) | .069 | 1 | .793 |
|  |  | Agerecoded(3) | .599 | 1 | .439 |
|  |  | Agerecoded(4) | .006 | 1 | .941 |
|  |  | levelofcreatinineafterthestartofRx(1) | 3.643 | 1 | .056 |
|  | Overall Statistics | | 33.061 | 12 | .001 |

**Block 1: Method = Enter**

| **Omnibus Tests of Model Coefficients** | | | | |
| --- | --- | --- | --- | --- |
|  | | Chi-square | df | Sig. |
| Step 1 | Step | 36.593 | 12 | .000 |
|  | Block | 36.593 | 12 | .000 |
|  | Model | 36.593 | 12 | .000 |

| **Model Summary** | | | |
| --- | --- | --- | --- |
| Step | -2 Log likelihood | Cox & Snell R Square | Nagelkerke R Square |
| 1 | 202.402^a^ | .180 | .248 |
| a. Estimation terminated at iteration number 5 because parameter estimates changed by less than .001. | | | |

| **Classification Table^a^** | | | | | |
| --- | --- | --- | --- | --- | --- |
|  | Observed | | Predicted | | |
|  |  |  | outcome status | | Percentage Correct |
|  |  |  | good outcome | poor outcome |  |
| Step 1 | outcome status | good outcome | 103 | 16 | 86.6 |
|  |  | poor outcome | 35 | 30 | 46.2 |
|  | Overall Percentage | |  |  | 72.3 |
| a. The cut value is .500 | | | | | |

| **Variables in the Equation** | | | | | | | | | |
| --- | --- | --- | --- | --- | --- | --- | --- | --- | --- |
|  | | B | S.E. | Wald | df | Sig. | Exp(B) | 95% C.I.for EXP(B) | |
|  |  |  |  |  |  |  |  | Lower | Upper |
| Step 1^a^ | recentantimicrobialusehistory(1) | 1.050 | .390 | 7.252 | 1 | .007 | 2.857 | 1.331 | 6.135 |
|  | Typescomorbidity |  |  | 8.026 | 2 | .018 |  |  |  |
|  | Typescomorbidity(1) | 1.243 | .548 | 5.147 | 1 | .023 | 3.464 | 1.184 | 10.135 |
|  | Typescomorbidity(2) | .338 | .559 | .365 | 1 | .545 | 1.402 | .469 | 4.197 |
|  | URTIs(1) | 1.309 | .657 | 3.975 | 1 | .046 | 3.702 | 1.022 | 13.406 |
|  | respiratoryrate(1) | .897 | .359 | 6.235 | 1 | .013 | 2.452 | 1.213 | 4.956 |
|  | typeofpneumonia |  |  | .299 | 2 | .861 |  |  |  |
|  | typeofpneumonia(1) | -.169 | .383 | .194 | 1 | .660 | .845 | .399 | 1.790 |
|  | typeofpneumonia(2) | .129 | .651 | .039 | 1 | .843 | 1.138 | .317 | 4.078 |
|  | Agerecoded |  |  | 4.064 | 4 | .397 |  |  |  |
|  | Agerecoded(1) | 1.392 | .877 | 2.522 | 1 | .112 | 4.024 | .722 | 22.433 |
|  | Agerecoded(2) | 1.057 | .902 | 1.374 | 1 | .241 | 2.878 | .491 | 16.865 |
|  | Agerecoded(3) | .321 | 1.180 | .074 | 1 | .785 | 1.379 | .136 | 13.935 |
|  | Agerecoded(4) | 1.426 | 1.098 | 1.687 | 1 | .194 | 4.163 | .484 | 35.821 |
|  | levelofcreatinineafterthestartofRx(1) | .863 | .401 | 4.624 | 1 | .032 | 2.370 | 1.079 | 5.204 |
|  | Constant | -3.875 | 1.097 | 12.480 | 1 | .000 | .021 |  |  |
| a. Variable(s) entered on step 1: recentantimicrobialusehistory, Typescomorbidity, URTIs, respiratoryrate, typeofpneumonia, Agerecoded, levelofcreatinineafterthestartofRx. | | | | | | | | | |
