# Supplementary material for: Evaluation of current practice of antimicrobial use and clinical outcome of patients with pneumonia at a tertiary care hospital in Ethiopia: A prospective observational study
Source: PLoS One. 2020 Jan 30;15(1):e0227736. doi: 10.1371/journal.pone.0227736 (PMC6992215; doi:10.1371/journal.pone.0227736)
Supplement: S3 File — (DOCX) [file pone.0227736.s003.docx]

| VARIABLES | | | | | | | | | | Total | Good Outcome (n=129) | Poor Outcome (n=71) |  |
| --- | --- | --- | --- | --- | --- | --- | --- | --- | --- | --- | --- | --- | --- |
| **Age Category** | | | | | | | | | | | | | |
| <18 years | | | | | | | | | | 12 | 10 | 2 |  |
| 18-39 | | | | | | | | | | 99 | 60 | 39 |  |
| 40-64 | | | | | | | | | | 62 | 40 | 22 |  |
| 65-74 | | | | | | | | | | 16 | 12 | 4 |  |
| >=75 | | | | | | | | | | 11 | 7 | 4 |  |
| **Sex** | | | | | | | | | | | | | |
| Males | | | | | | | | | | 104 | 64 | 40 |  |
| Female | | | | | | | | | | 96 | 65 | 31 |  |
| **Comorbidities** | | | | | | | | | | | | | |
| Heart Failure | | | | | | | Yes | | | 42 | 32 | 10 |  |
|  |  |  |  |  |  |  | No | | | 158 | 97 | 61 |  |
| Hypertension | | | | | | | Yes | | | 23 | 17 | 6 |  |
|  |  |  |  |  |  |  | No | | | 177 | 112 | 65 |  |
| Diabetes | | | | | | | Yes | | | 23 | 16 | 6 |  |
|  |  |  |  |  |  |  | No | | | 178 | 113 | 65 |  |
| CHD | | | | | | | Yes | | | 17 | 14 | 3 |  |
|  |  |  |  |  |  |  | No | | | 183 | 115 | 68 |  |
| Malignancy | | | | | | | Yes | | | 83 | 45 | 38 |  |
|  |  |  |  |  |  |  | No | | | 117 | 84 | 33 |  |
| CLD | | | | | | | Yes | | | 2 | 1 | 1 |  |
|  | | | | | | | No | | | 198 | 128 | 70 |  |
| Stroke (new & old): | | | | | | | Yes | | | 13 | 10 | 3 |  |
|  |  |  |  |  |  |  | No | | | 187 | 119 | 68 |  |
| CPD | | | | | Yes | | | | | 29 | 17 | 12 |  |
|  |  |  |  |  | No | | | | | 171 | 112 | 59 |  |
| CKD | | | | | Yes | | | | | 14 | 8 | 6 |  |
|  |  |  |  |  | No | | | | | 186 | 121 | 65 |  |
| CNS disorder | | | | | Yes | | | | | 9 | 7 | 2 |  |
|  |  |  |  |  | No | | | | | 191 | 122 | 69 |  |
| Immunosuppression | | | | | Yes | | | | | 78 | 43 | 35 |  |
|  | | | | | No | | | | | 122 | 86 | 36 |  |
| Number of comorbidity | | | | | | | | | | | | | |
| One comorbidity | | | | | | | | | | 132 | 86 | 46 |  |
| Two comorbidity | | | | | | | | | | 43 | 27 | 16 |  |
| Three or more comorbidity | | | | | | | | | | 25 | 16 | 9 |  |
| Type of comorbidity | | | | | | | | | | | | | |
| Pneumonia with heart failure | | | | | | | | | | 40 | 30 | 10 |  |
| Pneumonia with Cancer | | | | | | | | | | 83 | 44 | 38 |  |
| Pneumonia without heart failure and cancer | | | | | | | | | | 78 | 55 | 23 |  |
| **Types of pneumonia** | | | | | | | | | | | | | |
| HAP | | | | | | | | | | 96 | 55 | 41 |  |
| CAP | | | | | | | | | | 83 | 58 | 25 |  |
| Others | | | | | | | | | | 21 | 16 | 5 |  |
|  | | | | | | | | | |  |  |  |  |
| **Risk Factors for pneumonia** | | | | | | | | | | | | | |
| Cigarette Smoking | | | | | | | | | Yes | **13** | **8** | **5** |  |
|  |  |  |  |  |  |  |  |  | No | 187 | 121 | 66 |  |
| URTIs | | | | | | | | | Yes | 14 | 5 | 9 |  |
|  |  |  |  |  |  |  |  |  | No | 186 | 124 | 62 |  |
| Immunosuppressive agents | | | | | | | | | Yes | 78 | 23 | 13 |  |
|  |  |  |  |  |  |  |  |  | No | 164 | 106 | 58 |  |
| Old Age | | | | | | | | | Yes | 25 | 18 | 7 |  |
|  | | | | | | | | | No | 175 | 111 | 64 |  |
| Pre-existing LD | | | | | | | | | Yes | 37 | 19 | 18 |  |
|  | | | | | | | | | No | 163 | 110 | 53 |  |
| Other Chronic Diseases | | | | | | | | | Yes | 182 | 114 | 68 |  |
|  | | | | | | | | | No | 18 | 15 | 3 |  |
| Microbiological Information | | | | | | | | | | | | | |
| Microbiological studies: | | | | | | | | Yes | | 75 | 47 | 28 |  |
|  | | | | | | | | No | | 125 | 82 | 43 |  |
| **Source o sample** | | | | | | | | | | | | | |
| Blood | | | | | | | | | | 48 | 31 | 17 |  |
| Pleural Fluid | | | | | | | | | | 10 | 5 | 5 |  |
| Sputum | | | | | | | | | | 7 | 5 | 2 |  |
| Others | | | | | | | | | | 10 | 6 | 4 |  |
| Microbial Growth | | | | Yes | | | | | | 10 | 9 | 1 |  |
|  | | | | No | | | | | | 65 | 38 | 27 |  |
| **Time of culture collection** | | | | | | | | | | | | | |
| Day 1 | | | | | | | | | | 0 | 0 | 0 |  |
| Day 2 | | | | | | | | | | 2 | 2 | 0 |  |
| Day 3 | | | | | | | | | | 15 | 8 | 7 |  |
| Day 4 | | | | | | | | | | 8 | 5 | 3 |  |
| Day 5 | | | | | | | | | | 5 | 2 | 3 |  |
| After day 5 | | | | | | | | | | 45 | 30 | 15 |  |
| **Cause of microbiological test not to be done (Reason of missing the test)** | | | | | | | | | | | | | |
| No institutional guidance | | | | | | | | | | 20 | 10 | 10 |  |
| No well-equipped microbiological lab | | | | | | | | | | 8 | 6 | 2 |  |
| It is usual practice | | | | | | | | | | 61 | 44 | 17 |  |
| The patient has already started antimicrobials | | | | | | | | | | 22 | 13 | 9 |  |
| **Initial antimicrobial Therapy** | | | | | | | | | | | | | |
| Empiric | | | | | | | | | | 199 | 128 | 71 |  |
| Definitive | | | | | | | | | | 1 | 1 | 0 |  |
| Other reasons | | | | | | | | | | 14 | 9 | 5 |  |
| **Time of antimicrobial Initiation** | | | | | | | | | | | | | |
| Within 24 hours | | | | | | | | | | 127 | 79 | 48 |  |
| After 24 hours | | | | | | | | | | 73 | 50 | 23 |  |
| Is there missed dose during the course of treatment? | | | | | | Yes | | | | 60 | 39 | 21 |  |
|  |  |  |  |  |  | No | | | | 140 | 90 | 50 |  |
| Is there antimicrobial changes | | | | | | Yes | | | | 83 | 47 | 36 |  |
|  |  |  |  |  |  | No | | | | 117 | 82 | 35 |  |
| **Reason for first time change** | | | | | | | | | | | | | |
| Poor Response | | | | | | | | | |  | 12 | 13 |  |
| Inadequate selection (need for broader coverage) | | | | | | | | | | 11 | 8 | 3 |  |
| Side effect of antimicrobials | | | | | | | | | | 6 | 3 | 3 |  |
| Drug shortage/stock outs | | | | | | | | | | 18 | 8 | 10 |  |
| Due to discharge | | | | | | | | | | 2 | 1 | 1 |  |
| Change in diagnosis (within pneumonia) | | | | | | | | | | 13 | 9 | 4 |  |
| Other reasons | | | | | | | | | | 8 | 6 | 2 |  |
| **Antimicrobial Treatment duration** | | | | | | | | | | | | | |
| >=14 days | | | | | | | | | | 73 | 44 | 29 |  |
| <14days | | | | | | | | | | 127 | 85 | 42 |  |
| Eligibility of patients for IV to PO conversion | | | | | | Yes | | | | 110 | 97 | 13 |  |
|  |  |  |  |  |  | No | | | |  | 32 | 58 |  |
| Is there IV to PO conversion | | | | | | Yes | | | |  | 4 | 0 |  |
|  |  |  |  |  |  | No | | | |  | 125 | 71 |  |
| **Clinical Outcome** | | | | | | | | | | | | | |
| Stable | | | | | | | | | | 129 | 129 | 0 |  |
| Mortality due to pneumonia | | | | | | | | | | 13 | 0 | 13 |  |
| All-cause mortality | | | | | | | | | | 24 | 0 | 24 |  |
| Complications/No improvement | | | | | | | | | | 34 | 0 | 34 |  |
| **Clinical parameters upon admission** | | | | | | | | | | | | | |
| Temperature≤35 or ≥37.8^o^C | | | Yes | | | | | | | 96 | 58 | 38 |  |
|  |  |  | No | | | | | | | 97 | 64 | 33 |  |
| SBP<90 or >140mmHg | | | Yes | | | | | | | 31 | 23 | 8 |  |
|  |  |  | No | | | | | | | 163 | 101 | 62 |  |
| Pulse Rate>100 beats/min | | | Yes | | | | | | | 93 | 59 | 34 |  |
|  |  |  | No | | | | | | | 100 | 65 | 35 |  |
| RR<12 or >24 br/min | | | Yes | | | | | | | 93 | 51 | 42 |  |
|  |  |  | No | | | | | | | 101 | 73 | 28 |  |
| SaO_2_<90% | | | Yes | | | | | | | 107 | 66 | 41 |  |
|  |  |  | No | | | | | | | 86 | 57 | 29 |  |
| **Laboratory findings** | | | | | | | | | | | | |  |
| Level of BUN | | **<=**20mg/dl | | | | | | | | 66 | 50 | 16 |  |
|  |  | >20mg/dl | | | | | | | | 123 | 73 | 50 |  |
| S_cr_ before start of Rx | >1.4mg/dl | | | | | | | | | 31 | 19 | 12 |  |
|  | ≤1.3mg/dl | | | | | | | | | 158 | 104 | 54 |  |
| S_cr_ after start of Rx | >1.4mg/dl | | | | | | | | | 49 | 27 | 22 |  |
|  | ≤1.3mg/dl | | | | | | | | | 140 | 96 | 44 |  |
| **Radiologic Studies** | | | | | | | | | | | | | |
| CXR | | | | | | | | | | 91 | 63 | 28 |  |
| Chest CT | | | | | | | | | | 24 | 12 | 12 |  |
| Other (ultrasound) | | | | | | | | | | 6 | 4 | 2 |  |
| **Findings** | | | | | | | | | |  |  |  |  |
| Normal | | | | | | | | | | 9 | 4 | 5 |  |
| Confirmed pneumonia | | | | | | | | | | 67 | 48 | 19 |  |
| Other findings | | | | | | | | | | 45 | 27 | 18 |  |
|  | | | | | | | | | | | | | |
|  | | | | | | | | | |  |  |  |  |
|  | | | | | | | | | |  |  |  |  |
|  | | | | | | | | | |  |  |  |  |
|  | | | | | | | | | |  |  |  |  |
|  | | | | | | | | | |  |  |  |  |
|  | | | | | | | | | |  |  |  |  |
